# What mediates the racial/ethnic disparity in psychosocial stress among breast cancer patients?

**DOI:** 10.1007/s10552-021-01392-7

**Published:** 2021-02-09

**Authors:** C. T. Sánchez-Díaz, S. Strayhorn, S. Tejeda, G. Vijayasiri, G. H. Rauscher, Y. Molina

**Affiliations:** 1 Division of Epidemiology and Biostatistics, School of Public Health, University of Illinois at Chicago, 1603 West Taylor Street, Chicago, IL 60612 USA; 2 Division of Community Health Sciences, School of Public Health, University of Illinois at Chicago, Chicago, USA; 3 Institute for Health Research and Policy, University of Illinois at Chicago, Chicago, USA

**Keywords:** Racial/ethnic disparities, Psychosocial stress, Breast cancer

## Abstract

**Background:**

Prior studies have observed greater levels of psychosocial stress (PSS) among non-Hispanic (nH) African American and Hispanic women when compared to nH White patients after a breast cancer diagnosis. We aimed to determine the independent and interdependent roles of socioeconomic position (SEP) and unmet support in the racial disparity in PSS among breast cancer patients.

**Methods:**

Participants were recruited from the Breast Cancer Care in Chicago study (*n* = 989). For all recently diagnosed breast cancer patients, aged 25–79, income, education, and tract-level disadvantage and affluence were summed to create a standardized socioeconomic position (SEP) score. Three measures of PSS related to loneliness, perceived stress, and psychological consequences of a breast cancer diagnosis were defined based on previously validated scales. Five domains of unmet social support needs (emotional, spiritual, informational, financial, and practical) were defined from interviews. We conducted path models in MPlus to estimate the extent to which PSS disparities were mediated by SEP and unmet social support needs.

**Results:**

Black and Hispanic patients reported greater PSS compared to white patients and greater unmet social support needs (*p* = 0.001 for all domains). Virtually all of the disparity in PSS could be explained by SEP. A substantial portion of the mediating influence of SEP was further transmitted by unmet financial and practical needs among Black patients and by unmet emotional needs for Hispanic patients.

**Conclusions:**

SEP appeared to be a root cause of the racial/ethnic disparities in PSS within our sample. Our findings further suggest that different interventions may be necessary to alleviate the burden of SEP for nH AA (i.e., more financial support) and Hispanic patients (i.e., more emotional support).

## Introduction

Receiving a breast cancer diagnosis can lead to psychological social stress (PSS; e.g., negative perceptions about illness consequences, distress, loneliness), which has been associated with worse health-related quality of life (QOL), physiological side effects, and treatment decisions [1, 2]. Non-Hispanic African American (nH AA) and Hispanic women experience greater PSS and adverse consequences associated with breast cancer, when compared to non-Hispanic White (nH White) women in the USA [3–11].

Socio-ecological models are among the most popular theoretical frameworks for understanding racial/ethnic disparities in breast cancer and include multiple potential mechanisms at different levels [12, 13]. For the current study, we focus on two major, well-studied determinants—low socioeconomic position (SEP) and unmet social support needs. Here, SEP is defined according to patients’ level of resources and prestige when compared to others and is conceptualized as a wide-ranging concept that can be assessed at the individual, household unit, neighborhood, or community [14, 15]. Social support is defined as a positive interaction wherein patients are helped by someone within their network [16]. These potential determinants are often treated analytically as independent, sometimes competing mediators [9–11, 17]. Existing work that has considered SEP and unmet social support needs simultaneously has been mixed, with some studies suggesting that unmet social support needs explain more of racial/ethnic cancer disparities [10], other work suggesting SEP explains more of racial/ethnic disparities [11], and other work has suggested both are important [17].

One reason for conflicting findings may be interdependent effects, posited by several theoretical frameworks. Specifically, SEP is theorized to be a root cause of disparities, wherein it may directly affect BC outcomes *and* indirectly influence them through other determinants, including unmet social support [13, 18]. Through this theoretical lens, racial/ethnic minorities are subject to low SEP due to historic and contemporary systemic marginalization. Disparities in SEP may lead to racial/ethnic minorities’ disproportionate exposure to challenging interpersonal dynamics within medical settings (e.g., access to limited and inadequate support services) and community contexts (e.g., overburdened social networks, multiple completing support needs). Lower SEP and associated greater exposure to under resourced environments may consequently lead to lower perceived social support among racial/ethnic minorities, resulting in worse PSS.

Little to no research has quantified the interdependence between these mediating factors on racial/ethnic disparities in breast cancer.

Overall, we need research that expands on past research and examines the relative independent and interdependent effects of co-occurring, important mechanisms of racial disparities in breast cancer. To address this need, in the present study, we used population-based data from the Breast Cancer Care in Chicago study to evaluate the role of SEP and five domains of social support in explaining racial/ethnic disparities in PSS among breast cancer survivors. Specifically, we will address this gap by examining the independent and interdependent mediating paths by which SEP and unmet social support needs contribute to racial/ethnic disparities in breast cancer.

## Methods

The Breast Cancer Care in Chicago (BCCC) was a population-based, cross-sectional study of newly diagnosed breast cancer patients (*n* = 989). The study has been described in detail elsewhere [10, 19, 20]. Eligible BCCC participants were women between 25 and 79 years of age at diagnosis, who self-identified as nH white (*n* = 397), nH AA (*n* = 411), or Hispanic (*n* = 181), resided in Chicago and were diagnosed with a first primary breast cancer (in situ or invasive) between 2005 and 2008 (*n* = 981). The study received approval from the Institutional Review Board at the University of Illinois at Chicago and the Illinois Department of Public Health.

## Measures

### Psychosocial stress (PSS)

PSS was defined based on three existing validated scales. Four items from the Cohen Perceived Stress Subscale were summed to create a continuous perceived stress measure with an inter-item reliability (Cronbach’s alpha) = of 0.74) (0.81, 0.71, 0.64 for nH White, nH NH AA and Hispanic patients, respectively) [21, 22]. Three items from the UCLA Felt Loneliness Scale (Cronbach’s alpha  = 0.79) (0.79, 0.76 and 0.83 nH White, nH AA and Hispanic, respectively) [23], and 12 items from the Cockburn Psychological Consequences Scale (Cronbach’s alpha  = 0.93)) (0.79, 0.76 and 0.83 for nH White, nH AA and Hispanic, respectively) were also summed [24] to define additional measures of PSS. For descriptive analysis, each PSS measure was dichotomized at the sample median.

### Socioeconomic position (SEP)

SEP was defined using: (1) educational completion defined in years; (2) annual household income; and (3) two measures of SEP based on each woman’s census tract of residence (concentrated disadvantage or concentrated affluence) [19]. Concentrated disadvantage was defined as the percentage of families in the census tract with incomes below the poverty line; percentage of families receiving public assistance; percentage of persons unemployed; and percentage of female-headed households with children. Concentrated affluence was measured by percentage of families with incomes of $75,000 or more; percentage of adults with a college education or more; and percentage of the civilian labor force in professional and managerial occupations. Each measure of disadvantage and affluence was defined by creating an equally weighted sum across the relevant variables, then standardizing the sum to have a mean of 0 and a standard deviation of 1 [10–12, 25].

### Social support

We considered five specific domains of social support: emotional, spiritual, informational, financial, and practical [16]. *Emotional* support is defined here as providing patients with empathy, love and trust. *Spiritual* support is defined as providing patients with religious- or faith-based care. *Financial* support is defined as providing patients with financial/economic assistance. *Informational* support is defined as providing patients with necessary, helpful information. During the interview, the following script was read: “The next section of our interview concerns: help and support. People often need help or support when they have serious health problems. I am going to ask you some questions about how much help or support you needed and received since you were diagnosed with breast cancer.” Patients were first asked, “Since you were diagnosed with breast cancer, how much emotional help or support have you needed? Would you say none, a little, some, or a great deal?” This question was followed with, “How much emotional help or support have you received, from anyone?” Women rated how much help or support they needed (1 = none, 4 = a great deal) and how much they received since their diagnosis for the five specific domains. The difference between needed and received support was calculated to define a variable representing *unmet social support* for each of the five specific areas. These variables were then categorized as greater than zero (presence of unmet support need) and less than or equal to zero (absence of unmet support need) [10].

### Participant characteristics

Race/ethnicity was self-reported as nH White, nH AA and Hispanic. Age at diagnosis was defined in years and categorized as < 50, 50–59 and 60–79 for descriptive analyses. Stage at diagnosis was categorized into American Joint Committee on Cancer categories of 0, I, II, III, and IV. Binary variables for initiation of chemotherapy, radiation and hormone therapy were defined for this study from a combination of self-reports and medical records [26].

### Statistical analysis

#### Descriptive analysis

We tabulated the distribution of patient SEP and clinical factors by race/ethnicity (Table 1) and associations with measures of psychological stress (Table 2). We also obtained *p* values from a chi-square tests of association for nominal covariates and from a test for trend for ordered covariates. We then estimated Y-standardized linear regression coefficients for the age-adjusted association of the three psychosocial stress measures comparing nH AA versus nH White and again comparing Hispanic versus nH White. Coefficients were Y-standardized in order to be able to make fair comparisons regarding the magnitude of associations between PSS measures (Table 3).

**Table 1:** Racial/ethnic differences in social support, sociodemogaphic, and clinical factors

	*N*	nH White (*n* = 397)	nH Black (*n* = 411)	Hispanic (*n* = 181)	*p* value
		%	%	%	
Age at diagnosis					
25–39	66	6	6	9	0.97
40–49	236	25	22	24	
50–59	307	31	31	32	
60–69	246	24	26	24	
70–79	134	14	15	10	
Education					
< 12	176	5	19	44	< 0.0001
12	193	13	25	22	
> 12	617	82	56	35	
Income					
< 20,000	262	11	38	38	< 0.0001
< 75,000	439	40	50	50	
> 75,000	259	49	13	12	
Insurance					
No outpatient insurance	128	6	15	25	< 0.001
Public insurance only	164	4	26	22	
Private	697	90	59	53	
Concentrated affluence					
< 1 SD below mean	65	1	10	11	< 0.0001
Within 1 SD of mean	729	59	83	85	
> 1 SD above mean	193	40	7	4	
Concentrated disadvantage					
< 1 SD below mean	142	31	1	7	< 0.0001
Within 1 SD of mean	652	67	56	87	
> 1 SD above mean	57	7	43	3	
Stage					
0	200	27	23	16	< 0.0001
1	289	38	31	30	
2	255	26	31	36	
4	43	3	5	5	
Initiated radiation					
No	484	43	56	46	< 0.01
Yes	503	57	44	54	
Initiated chemotherapy					
No	573	67	52	52	< 0.0001
Yes	414	33	48	48	
Initiated hormone therapy					
No	711	69	77	68	0.01779
Somewhat/not at all	171	11	19	27	
Unmet emotional support					
No	905	95	90	87	< 0.0001
Yes	84	5	10	13	
Unmet spiritual support					
No	861	87	90	81	0.009
Yes	127	13	10	19	
Unmet informational support					
No	857	91	85	81	< 0.001
Yes	132	9	15	19	
Unmet financial support					
No	721	84	64	69	< 0.0001
Yes	268	16	36	31	
Unmet practical support					
No	871	93	84	86	< 0.0001
Yes	118	7	16	14

**Table 2:** Differences in the prevalence of self-reported psychosocial stress, defined as reporting values above the sample median, by sociodemogaphic and clinical factors

	Loneliness		Perceived stress		Psychological consequences	
	%	*p* value	%	*p* value	%	*p* value
Race/ethnicity		0.0009		< 0.0001		0.075
nH white	28		38		41	
nH black	37		48		44	
Hispanic	43		60		51	
Age at diagnosis		0.05		< 0.0001		< 0.0001
25–39	46		58		68	
40–49	35		54		53	
50–59	38		52		50	
60–69	33		38		34	
70–79	24		28		20	
Education		0.00005		< 0.0001		0.06
< 12	47		63		49	
12	37		45		46	
> 12	30		42		42	
Income		< 0.0001		< 0.0001		0.02
< 20,000	50		58		51	
< 75,000	38		47		43	
> 75,000	17		33		40	
Insurance		< 0.0001		< 0.0001		< 0.00001
No outpatient insurance	46		66		59	
Public insurance only	46		59		51	
Private	30		39		39	
Concentrated disadvantage		0.03		0.02		
< 1 SD below mean	25		34		42	
Within 1 SD of mean	36		48		44	
> 1 SD above mean	38		48		45	
Concentrated affluence		0.002		0.01		
< 1 SD below mean	38		48		46	
Within 1 SD of mean	37		48		44	
> 1 SD above mean	24		36		42	
Stage at diagnosis				0.002		< 0.0001
0	34		42		35	
1	35		43		38	
2	36		51		55	
3/4	39		58		55	
Initiated radiation						
No	37		47		44	
Yes	33		45		43	
Initiated chemotherapy				0.0003		< 0.0001
No	34		41		35	
Yes	36		53		56	
Initiated hormone therapy				0.051		0.0007
No	34		48		47	
Yes	36		41		35	
Unmet emotional support		< 0.0001		< 0.0001		< 0.0001
No	31		44		41	
Yes	79		73		73	
Unmet spiritual support		< 0.0001		< 0.0001		< 0.0001
No	31		44		41	
Yes	57		61		67	
Unmet informational support		< 0.0001		0.001		< 0.0001
No	32		44		41	
Yes	56		59		63	
Unmet financial support		< 0.0001		< 0.0001		< 0.0001
No	30		39		38	
Yes	49		64		60	
Unmet practical support		< 0.0001		< 0.0001		< 0.0001
No	31		43		41	
Yes	63		71		67

**Table 3:** Associations between race/ethnicity and PSS before and after adjusting for SES

	Adjusted for	Beta(STDY)^1^	*p* value
Loneliness			
nH AA	Age	0.1133	0.001
nHAA	Age, SES	− 0.0629	0.16
Hispanic	Age	0.1531	0.0000
Hispanic	Age, SES	0.0221	0.58
Stress			
nH AA	Age	0.0985	0.0040
nHAA	Age, SES	− 0.0226	0.61
Hispanic	Age	0.1485	0.0000
Hispanic	Age, SES	0.0585	0.14
Consequences			
nH AA	Age	0.0956	0.006
nHAA	Age, SES	− 0.0089	0.85
Hispanic	Age	0.1110	0.001
Hispanic	Age, SES	0.0344	0.40

#### Path models

**Fig. 1:**
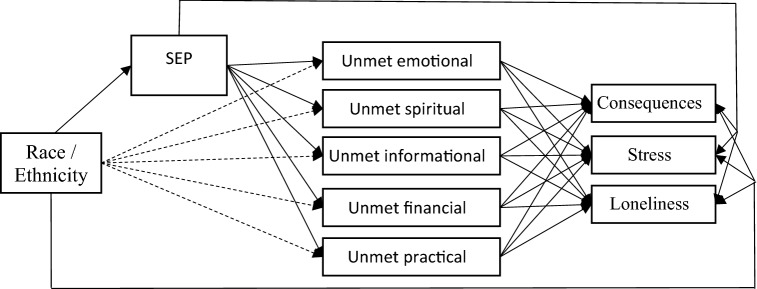
Path diagram corresponding to the path analysis. Dashed arrows represent direct associations for race/ethnicity with unmet support needs not mediated by SEP. Race/ethnicity was specified as two indicator variables for nH AA and Hispanic (nH white as referent)

Path models were estimated using Mplus, version 8 [27] to examine the mediating role of unmet social support needs and SEP in explaining the racial disparities in all three measures of PSS. Direct associations between race/ethnicity and each outcome were estimated along with all indirect associations through unmet social support needs and SEP. The figure below represents the path diagram corresponding to the structural equation model. Probit models for binary dependent variables (unmet social support) and linear regressions for continuous PSS dependent variables were estimated using full information maximum likelihood to account for missing data (MAR assumption). All models were adjusted for age (not shown in Fig. 1).

Each of the three continuous PSS variables were modeled as dependent variables in linear regression against unmet social support variables, continuous SEP, indicator variables for nH AA and Hispanic race/ethnicity, and age (continuous). Each of the five unmet support needs variables were modeled as dependent variables in probit regression against continuous SEP, indicator variables for nH AA and Hispanic race/ethnicity, and age. SEP was modeled in linear regression as a dependent variable against indicator variables for nH AA and Hispanic race/ethnicity, and age.

Indirect associations for racial/ethnic disparities in PSS variables as mediated by unmet support needs and SEP were obtained by taking the product of coefficients within each path from race/ethnicity to specific PSS variable. We then calculated the proportion of the racial/ethnic disparities in PSS independently explained by unmet social support needs domains and SEP. The proportion mediated was independently calculated as the association of the indirect association divided by the total association. This was calculated for all possible indirect pathways. A mediation proportion of greater than 100% occurred when control for a mediator changed the sign of the coefficient of interest, which may have happened for substantive reasons or simply due to the typical instability of the resulting associations.

## Results

### Descriptive analysis

Table 1 shows racial/ethnic differences in sociodemographic and selected patient characteristics. Overall, mean age was similar for all racial/ethnic groups (56 ± 0.56, 57 ± 0.55 and 54 ± 0.86 for nH White, nH AA and Hispanics, respectively). nH AA were more likely to live in disadvantage when compared to nH Whites and Hispanic women (43.1% versus 1.16% and 6%, respectively), whereas Hispanic participants reported lower education and were more likely to be uninsured (44% less than high school and 25%uninsured) when compared to nH Whites (5% less than high school and 6% uninsured) and nH AA (19% less than high school and 15% uninsured) (*p* < 0.0001 and *p* < 0.001, respectively). Both nH AA (38%) and Hispanic (38%) women reported lower annual income when compared to nH Whites (11%) (*p* < 0.0001) (Table 1). In terms of unmet social support, nH AA and Hispanic patients were more likely to have unmet financial, emotional and practical support than nL Whites (*p* < 0.001). In addition, Hispanic patients were more likely to have unmet l, spiritual and informational support when compared to nH Whites (*p* < 0.001).

Overall, younger patients, racial/ethnic minorities and patients with lower socioeconomic position reported a higher prevalence of PSS (Table 2). Later stage at diagnosis was associated with greater perceived stress and greater psychological consequences (*p*_trend_ < 0.001 and < 0.0001, respectively). For each of the five measures of unmet support needs and for each of the three measures of PSS, greater unmet need was associated with greater PSS (Table 2).

Results from our Y-standardized linear models showed that for all three PSS measures and for both nH AA and Hispanic versus nH White patients, controlling for SES either eliminated or greatly attenuated associations of race/ethnicity with PSS (Table 3). For example, for loneliness among nH AA, the age-adjusted standardized Beta changed from β_STDY:_ 0.1133 (*p* < 0.001) to β_STDY_ − 0.0629 (*p* = 0.16) after including SES in the model.

### Path models

The model fit statistics for this path model indicated a good fitting model (CFI = 1.000, RMSEA = 0.036, *p* value from *χ*^2^ Test of Model Fit = 0.14). From this model, we first estimated indirect associations for SEP and unmet support needs, and the proportion of each disparities that was independently mediated by SEP and unmet support needs.

SEP accounted for most of the nH AA-nH White disparities in PSS, including in psychological consequences, perceived stress, and loneliness (proportion mediated ≥ 86%). There were no mediating effects of unmet social support needs on PSS that were independent of SEP. However, a large proportion of the statistical mediation by SEP was the result of further mediation by unmet financial and practical needs within the nH AA-nH White disparity (Table 4).

**Table 4:** Path model for socioeconomic status and psychosocial stress in mediating the nH Black and nH White disparity in psychosocial stress in the Breast Cancer Care in Chicago

	Pychological consequences			Perceived stress			Loneliness		
	Value	*p* value	*p* value	Value	*p* value	*p* value	Value	*p* value	*p* value
Total association (fully standardized)	0.110	0.003	0.001	0.085	0.016	0.02	0.110	0.003	0.001
Proportion mediated	95%	0.026	0.03	86%	0.014	0.01	164%	0.004	0.004
Through SES									
Overall	109%	0.034	0.03	102%	0.031	0.03	174%	0.007	0.007
*Via* unmet needs	52%	0.136	0.14	34%	0.055	0.06	125%	0.011	0.01
* Not via* unmet needs	57%	0.027	0.03	68%	0.032	0.03	49%	0.019	0.02
Through unmet needs									
Overall	43%	0.02	0.02	52%	0.022	0.02	39%	0.015	0.02
Through individual unmet needs									
Emotional	11%	0.295	0.30	11%	0.305	0.31	10%	0.307	0.31
Spiritual	3%	0.547	0.55	3%	0.548	0.55	1%	0.566	0.57
Informational	4%	0.225	0.23	− 3%	0.341	0.34	1%	0.606	0.61
Financial	17%	0.061	0.06	23%	0.057	0.06	13%	0.089	0.09
Practical	9%	0.116	0.12	19%	0.06	0.06	14%	0.042	0.04

Estimates and *p* values are suppressed when the proportion mediated is < 5% or the corresponding *p* value > 0.20

SEP similarly accounted for most of the Hispanic-nH White disparity in PSS, including 69% of the disparity in psychological consequences, 46% in perceived stress and 94% in loneliness. There were no mediating effects of unmet social support needs on PSS that were independent of SEP. However, a substantial portion of the statistical mediation by SEP was due to unmet emotional needs (Table 5). Finally, we ran a sensitivity analysis excluding women below 40y/o (*n* = 66), the age which women begin mammogram screening, and no differences were observed in our results (Data not shown).

**Table 5:** Path model for socioeconomic status and psychosocial stress in mediating the Hispanic and nH White disparity in psychosocial stress in the Breast Cancer Care in Chicago

	Pychological consequences			Perceived stress			Loneliness		
	Value	*p* value	*p* value	Value	*p* value	*p* value	Value	*p* value	*p* value
Total association (fully standardized)	0.112	0.001	0.001	0.138		< 0.0001	0.150		< 0.0001
Proportion mediated	80%	0.006	0.01	44%	0.002	0.002	98%	0	< 0.0001
Through SES									
Overall	69%	0.018	0.02	46%	0.003	0.003	94%	0	< 0.0001
*Via* unmet needs	33%	0.116	0.12	15%	0.014	0.01	67%	0.001	0.001
* Not via* unmet needs	36%	0.013	0.01	31%	0.003	0.003	26%	0.003	0.003
Through unmet needs			0.00						
Overall	47%	0.003	0.00	28%	0.012	0.01	31%	0.002	0.002
Through individual unmnt needs			0.00						
Emotional	25%	0.014	0.01	17%	0.013	0.01	19%	0.008	0.01
Spiritual	7%	0.123	0.12	5%	0.128	0.13	3%	0.206	0.21
Informational	8%	0.104	0.10	− 4%	0.229	0.23	1%	0.586	0.59
Financial	6%	0.139	0.14	6%	0.117	0.12	4%	0.169	0.17
Practical	3%	0.328	0.33	4%	0.297	0.30	4%	0.281	0.28

Estimates and *p* values are suppressed when the proportion mediated is < 5% or the corresponding *p* value > 0.20

## Discussion

Our study adds to a growing body of literature that directly quantifies underlying mechanisms of racial/ethnic disparities in PSS among breast cancer patients. We found that SEP mediated virtually all of the racial/ethnic disparity in our three PSS measures for both NHB and Hispanic patients. Part of this mediation of SEP was further mediated by unmet social support needs, but in a different way for NHB and Hispanic patients*.* Greater unmet financial and practical support needs each accounted for between roughly one-tenth and one-fifth of the NHB, NHW disparity across the three PSS measures; in contrast, greater unmet emotional support needs accounted for between roughly one-fifth to one-fourth of the Hispanic, NHW disparity in PSS.

Our first finding aligns with a large body of research clarifying that racial/ethnic minorities are particularly vulnerable to financial strain pre and post cancer diagnosis [18–22]. Specifically, our study found racial/ethnic differences in financial strain that are consistent with existing literature. Where, in general and pre-cancer diagnosis, Hispanic and nH AAs are more likely to have poor SEP at the individual (e.g., lack health insurance, have lower income, lower educational status) and neighborhood-level (e.g., medical deserts, concentrated poverty) when compared to nH Whites [28–30]. It is thus not surprising that non-Hispanic and nH AAs experience greater financial burden from cancer diagnoses than nH White women [31, 32]. Simultaneously, low SEP, especially when operationalized at multiple levels—i.e., composites that include both individual- and neighborhood-level aspects—has been associated with worse PSS and physical health outcomes [25, 33, 34].

Our novel approach clarified past conflicting research [9–11]. Specifically, existing work that has considered SEP and unmet social support needs simultaneously has been mixed, with some studies suggesting that unmet social support needs explain more of racial/ethnic cancer disparities [10], other work suggesting SEP explains more of racial/ethnic disparities [11], and other work has suggested both are important [17]. For example, two previous studies used our same data source, SEP, and unmet social support measures [10, 11]. Using the same dataset that we used, Tejeda et al. summed the 5 variables for support needed and again for support received then calculated the difference between needed and received support to create a continuous variable for unmet social support. Unmet support needs were the strongest mediator of racial/ethnic disparities in one of the PSS measures used in our study (psychological consequences after a BC diagnosis) [10]. Our study expanded on this study by tearing apart the five dimensions of unmet needs and the intertwined roles that SES and unmet needs play in mediating racial/ethnic disparities in PSS, and by including two additional measures of PSS. A second analyses of these data by Vijasayisiri et al. focused on BC-specific survival and did not examine PSS as either a dependent or independent variable. Neighborhood-concentrated disadvantage (one component of our SES measure) was associated with worse survival, whereas social network size and density, as well as practical and financial support were each associated with improved 5-year survival. Neighborhood-concentrated disadvantage and practical support each mediated a portion of the nH AA- nH White survival disparity.

On the other hand, the study by Thompson et al., used a smaller sample of *n* = 229 nH AA newly BC diagnosed women to evaluate: ((a) associations of initial levels of perceived social support with demographic and clinical factors and (b) associations of perceived social support with depressive symptoms after adjusting by individual level sociodemographic and clinical covariates [35]. Contrary to our study: (1) Thompson et al., evaluated changes in social support post diagnosis and after a 2 year follow-up period using the Medical Outcomes Study Social Support Survey (MOS-SSS). Similar to our findings, after including SES in their models, the association of perceived social support with depressive symptoms was attenuated, which is consistent with a role for SES in mediating the association of social support with depressive symptoms.

Our work highlights *how* SEP may result in lower PSS through unmet social support, in line with extant theoretical frameworks [11, 12, 14, 16]. Further, our consideration of different social support domains adds to a large, complicated picture of racial/ethnic differences in social support [10, 36–38] by clarifying the specific domains of support through which SEP results in PSS disparities. For nH AA patients, our work suggests interventions that provide explicit, concrete financial support may be particularly helpful to address patients’ objective needs and perceptions about available financial resources. For Hispanic patients, our findings suggest that financial toxicity may subsequently lead to unmet emotional support. This relationship may be understood in the context of family dynamics, wherein patients belong to a culture that values social collectivism and interdependence, and may perceive themselves to be unduly burdensome to their family and friends because of their cancer diagnosis and its associated costs [39]. Notably, neither informational support nor spiritual support displayed an independent or interdependent mediating effect on the association between SEP and PSS.

This secondary data analysis has some limitations. First, we had a relatively small sample size, specifically for the Hispanic group, which may have impacted the precision of our estimates. Second, due to the cross-sectional design SEP, unmet social support needs and PSS were measured at the point in time post diagnosis. We assumed a causal ordering in our analyses such that SEP preceded unmet social support needs which preceded PSS. While these assumptions are theoretically reasonable, they were likely violated to an extent by our design. For example, SEP as measured might not reflect pre-diagnostic SEP if a diagnosis caused the loss of a job or other life event that created economic hardship affecting household income. Relatedly, while we assumed that unmet social support needs preceded PSS, increased levels of PSS resulting from a diagnosis could cause social network members to withdraw from the patients, producing an association due to reverse causality. Additionally, a patient experiencing high levels of PSS might perceive a higher level of unmet social support needs independent of the actual extent of support provided to her. A third limitation is the way we operationalized SEP, where we assumed equal impacts of contextual and individual level variables. However, contextual SEP theoretically predicts individual SES and may be more impactful as a root cause of unmet support needs and PSS. Our approach addresses an emergent need in the literature: future work should provide theory-driven guidance for multi-level SEP measurement (e.g., *how* and *when* to weight contextual versus individual level SES variables). Additionally, age categories were based on available sample characteristics. We ran a sensitivity where we excluded participants younger than 40y/o and we didn’t observe significant differences in the results from our path models. Fourth, future studies that distinguish between screening-eligible and younger populations are critical. Since women younger than 40 y/o are more likely to have children and may experience negative impacts of treatments on fertility—which has been associated to PSS. Finally, we used single-item measures for the different social support domains; while these measures have been previously used, there is a need to replicate our work with validated, commonly used multi-item measures (e.g., Medical Outcomes Study Social Support Survey MOS-SSS).

## Implications

Our findings are timely, given the increasing attention paid to developing financial support resources for cancer survivors overall [40–42]. Financial navigation and other types of interventions may be particularly useful for mitigating racial/ethnic disparities in PSS, given the large independent mediating role of SEP. Simultaneously, our work has the potential to help researchers develop prevention and intervention strategies, focusing in financial support for nHAA and in practical and emotional support for Hispanics. These, in turn, could be used to help eliminate cancer health disparities.

## Data Availability

For this study, the authors used Mplus 8 and Stata 15.1 for the analysis.

## Data availability

Data were obtained from the Breast Cancer Care in Chicago and the study was approved by the University of Illinois at Chicago Institutional Review Board.
